# The BUILT study: a single-center 5-year experience of Lung Cancer screening in Taiwan

**DOI:** 10.7150/ijms.64648

**Published:** 2021-10-31

**Authors:** Chih-Wei Wu, Yen-Te Ku, Chun-Yao Huang, Po-Chun Hsieh, Kun-Eng Lim, I-Shiang Tzeng, Chou-Chin Lan, Yao-Kuang Wu, Yi-Chiung Hsu

**Affiliations:** 1 Division of Pulmonary Medicine, Taipei Tzu Chi Hospital, Buddhist Tzu Chi Medical Foundation, New Taipei City, Taiwan.; 2 Department of Biomedical Sciences and Engineering, National Central University, Taoyuan, Taiwan.; 3 Department of Surgery, Taipei Tzu Chi Hospital, Buddhist Tzu Chi Medical Foundation, New Taipei City, Taiwan.; 4 Department of Chinese Medicine, Taipei Tzu Chi Hospital, Buddhist Tzu Chi Medical Foundation, New Taipei City, Taiwan.; 5 Department of Radiology, Taipei Tzu Chi Hospital, Buddhist Tzu Chi Medical Foundation, New Taipei City, Taiwan.; 6 Department of Research, Taipei Tzu Chi Hospital, Buddhist Tzu Chi Medical Foundation, New Taipei City, Taiwan.

**Keywords:** low dose CT, lung cancer, screening, pulmonary nodule

## Abstract

**Background:** There are no uniform guidelines on low-dose computed tomography (LDCT) follow-up in lung cancer screening. Few studies have analyzed the incidental abnormalities and role of tumor markers in lung cancer screening. The purpose of this study was to investigate the diagnostic performance of LDCT, optimal follow-up duration, incidental findings, and role of tumor markers in diagnosing lung cancer.

**Methods:** We retrospectively analyzed subjects who underwent their first LDCT in Taipei Tzu Chi Hospital between September 1, 2015, and August 31, 2016. All chest CT scans until August 31, 2020, were recorded. A non-calcified nodule with a diameter ≥2 mm on LDCT was defined as a positive result. We extracted the data, including possible risk factors of lung cancer and follow-up outcomes.

**Results:** A total of 1502 subjects were recruited. Of the 38 subjects who underwent biopsy, 31 had confirmed lung cancer. Lung cancer in all patients was diagnosed within 4 years. Univariate logistic regression analysis revealed that a family history of lung cancer in first-degree relatives and abnormal serum carcinoembryonic antigen (CEA) levels were the significant risk factors for lung cancer. A cumulative lung cancer incidence of 54.7 patients per 1000 person-years was determined solely via radiological follow-up. In total, 271 (18%) subjects exhibited incidental findings on baseline LDCT.

**Conclusion:** The overall lung cancer detection rate in this study was 2.1% in the 5-year study period. A family history of lung cancer and abnormal serum CEA levels are important risk factors for lung cancer. A minimum of 4-year follow-up is required to track suspicious nodules. A purely radiological follow-up detects a high incidence of lung cancer.

## Introduction

Lung cancer is the leading cause of cancer-related deaths worldwide, and it is estimated that 1.8 million patients (18% of all cancer deaths) died from lung cancer in 2020 1. According to the 2018 Taiwan Cancer Registry annual report, 16023 patients (13.2% of all cancers) had newly diagnosed lung cancer, and 9388 patients (19.2% of all cancers) died from lung cancer (i.e., 39.8 per 100,000 individuals) 2. In Taiwan, from 2014 to 2018, the 5-year survival rate of lung cancer was 28.6% 2. The prognosis of lung cancer is well correlated with its clinical or pathological stages 3. Most symptomatic patients with lung cancer have late-stage lung cancer 4. Lung cancer screening using low-dose computed tomography (LDCT) can identify asymptomatic patients with early stage lung cancer 5-8. In 2018, approximately 58% of patients with newly diagnosed lung cancer presented with stage III or IV disease in Taiwan 2.

The National Lung Screening Trial (NLST) and Nederlands-Leuvens Longkanker Screenings Onderzoek (NELSON) trial have proven that LDCT reduces lung cancer-related mortality in high-risk populations 5, 6. In the NLST, LDCT with a median 6.5-year follow-up showed an 18% reduction in lung cancer mortality and in the NELSON trial, a 10-year follow-up reduced lung cancer mortality by 26% in men and 39% in women. Long-term follow-up is difficult outside clinical trials. Thus, an applicable guideline for the duration of follow-up is needed.

LDCT requires more sophisticated techniques, is more expensive, and results in more radiation exposure to participants than conventional chest X-ray (CXR) 9. Thus, it is essential to maximize the utility of the LDCT. In addition to lung tumors, LDCT could provide valuable incidental findings, such as coronary artery calcification and emphysema 10. However, few studies have focused on these data. The role of tumor markers in lung cancer diagnosis remains unclear. Molina et al. showed that tumor markers have a potential role in lung cancer diagnosis 11. Tumor markers, such as carcinoembryonic antigen (CEA) and carbohydrate antigen 19-9 (CA19-9) are widely used in general health checkups 12.

In the present study, we aimed to investigate lung cancer incidence, risk factors of lung cancer, optimal follow-up length, and incidental LDCT findings in subjects who received general health examination in a single medical center of northern Taiwan during a 5-year follow-up.

## Materials and methods

**Figure 1:**
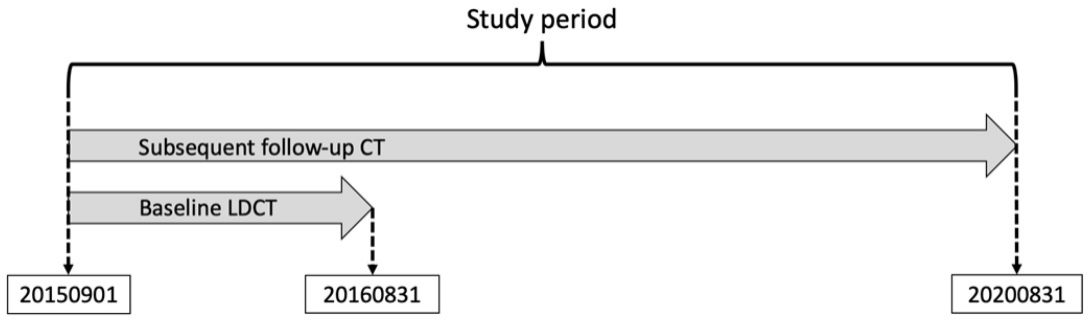
The study protocol. We enrolled subjects who received the baseline low-dose computed tomography (LDCT) scan from September 1, 2015 to August 31, 2016. The dates of all follow-up chest CT scans (LDCT or conventional CT) are recorded from September 1, 2015 to August 31, 2020.

The present **l**ung cancer screening study was conducted in Taipei Tzu Chi Hospital, **B**uddhist **T**zu Ch**i** Medical Foundation (**B**U**ILT** study). All participants were asymptomatic and underwent a self-funded health checkup, including LDCT and concomitant biomarker tests (CEA and CA 19-9) on the same day. We retrospectively recruited subjects who received their baseline LDCT scan between September 1, 2015 and August 31, 2016. CXR performed on the same day as the baseline LDCT was defined as baseline CXR. The follow-up chest CT scan included LDCT or conventional chest CT until August 31, 2020 (Figure 1). A serum level of CEA > 5 ng/mL or CA 19-9 > 37 U/mL was designated as a positive test.

Subjects with previous history of extrapulmonary malignancies were enrolled in the study. We excluded patients with a previous history of lung cancer, aged under 18 years, or those who had more than 10 lung nodules. The invasive diagnostic procedures used for histopathological examination in this study were surgery, bronchoscopy, or CT-guided biopsy. All subjects were divided into three groups: Group A, subjects who underwent biopsy and had pathologically confirmed lung cancer; Group B, subjects who underwent lung biopsy and had **b**enign pathology; Group C, subjects who received **c**onservative follow-ups without biopsy.

**Figure 2:**
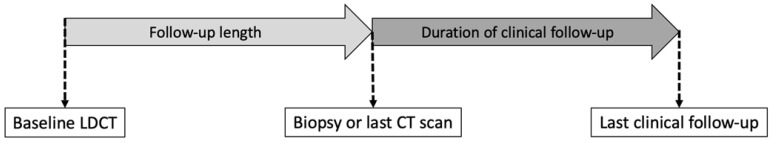
The definition of follow-up duration. In the BUILT study, the follow-up length is the time from the baseline low-dose computed tomography (LDCT) scan to biopsy (Group A, B) or the last CT scan (Group C). The duration of clinical follow-up is included in the total follow-up length of the NLST and NELSON trial, but **not** in the BUILT study.

The follow-up duration is shown in Figure 2. The follow-up duration in the BUILT study was the time from the baseline LDCT to biopsy (Group A, B) or the last CT scan (Group C). In the NLST and NELSON trial, clinical follow-ups were conducted after LDCT screening, including questionnaires or linkage to the national cancer database 5, 6. However, the duration of clinical follow-up was not included in the total follow-up duration of the present study, and this study design featured a purely radiological follow-up. For example, if the subject received only baseline LDCT scan during the study period, even with regular follow-up at the outpatient clinic, the follow-up duration was 1 day in the present study. We summed up all person-years of follow-up.

The time to diagnosis was the time between the baseline LDCT to biopsy (Group A, B) or the last CT scan (Group C). As a result, the follow-up length was the same as the time to diagnosis.

We reviewed the electronic medical records of all eligible subjects and extracted the data, including sex, age, smoking history, history of lung and extrapulmonary malignancy of first-degree relatives, number of nodules, a positive result in the baseline LDCT or CXR, number of CT scans until diagnosis, time to diagnosis, incidental findings in the baseline LDCT, biomarker tests, and comorbidities. The incidental findings included bronchiectasis, emphysema, fibrocalcified lesions compatible with old pulmonary tuberculosis, coronary artery calcifications, and extrapulmonary malignancy. The comorbidities included history of extrapulmonary malignancy, arrhythmia, heart failure, ischemic heart disease, old stroke, parkinsonism, hypothyroidism, hyperthyroidism, autoimmune disease, liver cirrhosis, chronic hepatitis, peptic ulcer, end-stage renal disease, asthma, chronic obstructive pulmonary disease, diabetes mellitus, and hypertension.

One radiologist and three pulmonologists (KL Lim, YK Wu, CC Lan, and CW Wu, with 30, 28, 25, and 12 years of experience in thoracic radiology, respectively) reviewed the nodule characteristics and incidental findings in the baseline LDCT and any suspicious nodules in the baseline CXR of the patients. The nodule characteristics included the size (the longest diameter in cross section), CT attenuation (ground glass opacity, part-solid, and solid), and nodule counts per scan. The extrapulmonary findings were assessed visually. A positive test during the baseline LDCT was defined as a noncalcified nodule with a longest diameter of ≥ 2 mm. A nodule with any suspicious features of malignancy was classified as a positive test in the baseline CXR.

The multidisciplinary Lung Cancer Committee of Taipei Tzu Chi Hospital comprises pulmonologists, thoracic surgeons, thoracic radiologists, and pathologists, and is responsible for the management of indeterminate nodules. The 8^th^ edition of the TNM stage classification published in 2016 was used to stage all proven lung cancers, except lymphoma 3.

LDCT scans were obtained using non-contrast, multi-detector CT (Brilliance iCT 256, Philips Healthcare, Cleveland, OH, USA). The basic parameters were 120-kilovoltage peak (tube voltage), 20 mAs (tube current-time product), 1.2 mSv (average radiation exposure), and 1.5 mm (collimation).

The study was approved by the Institutional Review Board of Taipei Tzu Chi Hospital, Buddhist Tzu Chi Medical Foundation (Protocol Number: 07-X-031), and the requirement for informed consent was waived.

### Statistical analysis

Categorical variables were analyzed using the chi-square test and are expressed as numbers (percentages). Continuous variables with non-Gaussian distribution were analyzed using the Mann-Whitney U test and are expressed as medians (1^st^ quartile - 3^rd^ quartile). Logistic regression was used to calculate odds ratios (ORs) with 95% confidence intervals (CI). Odds ratio was omitted in some results because small sample size leads to inflation or shrinkage of odds ratio (mentioned as Cannot-be-estimated instead). The time to diagnosis was plotted using the Kaplan-Meier method, and the difference was analyzed using the Breslow test. The diagnostic performance was evaluated by the area under the receiver operating characteristic (ROC) curve. Statistical significance was set at P < 0.05. IBM® SPSS® Statistics Version 24 software was used to analyze the data.

## Results

**Figure 3:**
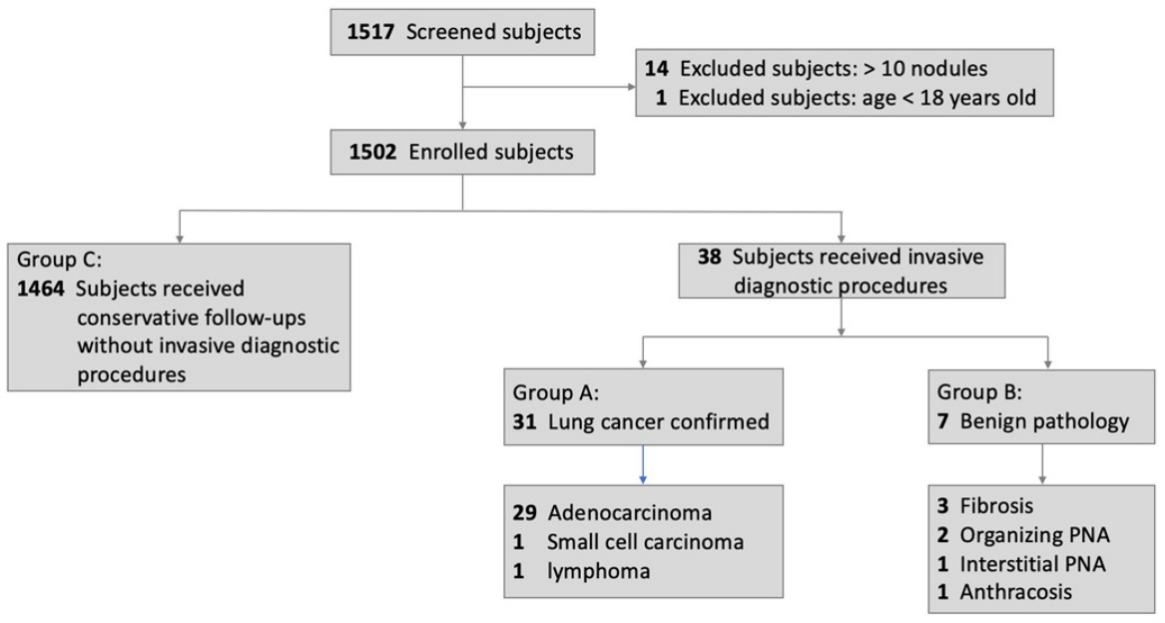
Flowchart of subjects' enrollment and diagnostic outcomes. Abbreviations: PNA, pneumonia.

Figure 3 shows the flowchart of the enrollment and diagnostic outcomes. A total of 1517 subjects were included in the study. Fourteen subjects were excluded because of the presence of more than 10 pulmonary nodules in the baseline LDCT scan, and one subject (17 years old) was excluded due to age < 18 years. Of the remaining 1502 subjects, 38 underwent biopsy, while 1464 (Group C) received conservative follow-ups. Of the 38 subjects who underwent biopsy, 31 (Group A) had pathologically confirmed lung cancers and 7 (Group B) had unnecessary resection of benign lung tumors (3 fibrosis, 2 organizing pneumonia, 1 interstitial pneumonia, 1 anthracosis).

Table 1 lists the baseline characteristics of all subjects and the possible risk factors for lung cancer. Subjects with confirmed lung cancer were included in Group A and those with non-confirmed lung cancer were classified as Group B + C. The first part of Table 1 compares the baseline characteristics of Group A and Group B + C. In total, 31 subjects (2.1%) had confirmed lung cancer. A total of 1471 (97.9%) subjects did not have lung cancer. Among the 1502 subjects, the percentages of smokers, ex-smokers, and never smokers were 2.8%, 6.0%, and 91.2%, respectively. Furthermore, 27 patients with confirmed lung cancer were never smokers. Among 78 subjects aged < 40 years, 1 had lung cancer; moreover, among 298 subjects aged < 50 years, 2 had lung cancer. There were no significant differences in sex, age, smoking history, family history of extrapulmonary cancer, incidental findings in the baseline LDCT, CA 19-9, and comorbidities between Group A and Group B + C. Nevertheless, Group A had a significantly higher percentage of family history of lung cancer, greater number of nodules, higher rate of positive results in the baseline LDCT and baseline CXR, greater number of chest CT scans until diagnosis, longer time to diagnosis, and higher rate of abnormal serum CEA than Group B + C. Of note, the incidental findings included an extrapulmonary malignancy (renal cell carcinoma) in Group B + C. The second part of Table 1 shows the logistic regression analysis of all possible risk factors for lung cancer. The significant risk factors included a family history of lung cancer, greater number of nodules, positive result in the baseline CXR, greater number of CT scans until diagnosis, longer time to diagnosis, and positive result for serum CEA level. Among the subjects with confirmed lung cancer (n = 31), 3 (9.7%) had abnormal serum CEA levels. Meanwhile, among subjects with abnormal serum CEA levels (n = 26), 3 (11.5%) had lung cancer. Positive serum CEA levels were significantly associated with the diagnosis of lung cancer (odds ratio = 6.745, 95% CI: 1.914-23.78).

Table 2 summarizes the cancer diagnosis and stage, epidermal growth factor receptor mutation status, radiologic features, treatments, and 3-year survival rates of patients with detected lung cancer. The pathology report included 25 invasive adenocarcinomas, 2 minimally invasive adenocarcinomas, 2 adenocarcinomas *in situ*, 1 small-cell carcinoma, and 1 mucosa-associated lymphoid tissue lymphoma (MALT lymphoma). The median time to diagnosis was 113 days. According to the 8^th^ edition of the American Joint Committee on Cancer (AJCC) staging system (lymphoma excluded), 26 patients (87%) had early stage lung cancer (stage 0 or I), 2 (6.7%) had stage III disease, and 2 (6.7%) had stage IV disease. Two patients (6.7%) had multiple primary lung cancers. Among the 31 patients with lung cancer, the 3-year progression-free survival rate and overall survival rate were 90.3% and 93.5%, respectively.

A total of 31 patients were detected with lung cancer per 566.6 person-years; thus, 54.7 patients were detected with lung cancer per purely radiological follow-up of 1000 person-years.

Table 3 illustrates the diagnostic performance of the baseline LDCT scan, the baseline CXR, and serum CEA and CA 19-9 levels. The baseline LDCT had the highest sensitivity (100%), negative predictive value (100%), and area under the ROC curve (0.885).

**Figure 4:**
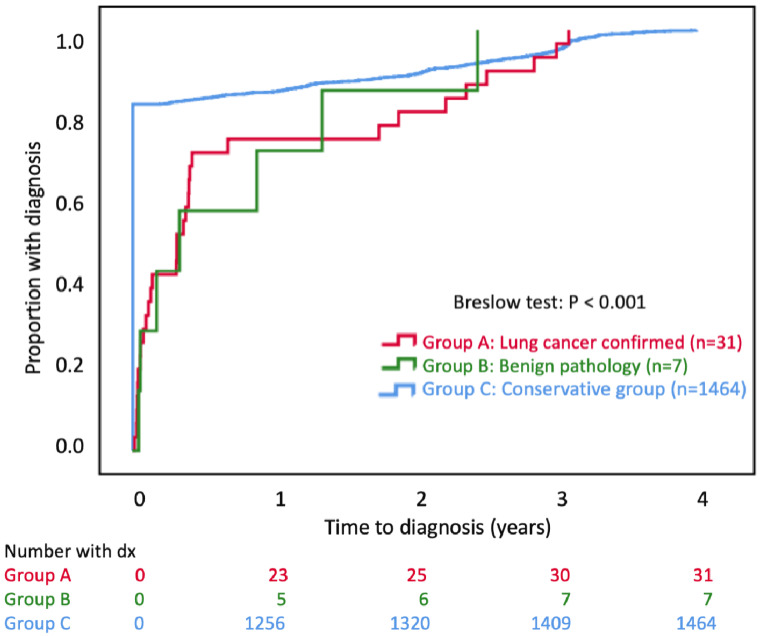
The Kaplan-Meier plot of cumulative proportions of subjects with diagnosis. The date of diagnosis in Group C is the date of the last CT scan during the study period. The date of diagnosis for Groups A or B is the biopsy date.

Figure 4 shows the Kaplan-Meier plot of the cumulative proportion with diagnosis. The time to diagnosis was significantly early in group C (P<0.001). All groups completed a 100% diagnosis within 4 years. The cumulative lung cancer detection rates in Group A were 1.5% (23/1502), 1.7% (25/1502), 2.0% (30/1502), and 2.1% (31/1502) by Year 1, Year 2, Year 3, and Year 4, respectively.

## Discussion

LDCT is effective in detecting lung cancer. Lung cancer was detected in 2.1% of the participants using LDCT in the 5-year BUILT study. Approximately 85% of lung cancers were diagnosed at a curable early stage with a 90% 3-year overall survival. All patients with lung cancer were detected within 4 years. A family history of lung cancer and abnormal serum CEA levels were significant risk factors for lung cancer. The “purely radiological” follow-up in the BUILT study detected a high cumulative incidence of lung cancer. Extrapulmonary abnormalities were detected in 18% of the participants. About one-fifth of the participants received unnecessary diagnostic procedures and had a benign pathology.

### Study strengths

The BUILT study had several strengths. First, we enrolled patients with previous history of extrapulmonary cancers, and a high proportion of participants were never-smokers. We searched for a detailed medical history and found that almost all participants had no significant comorbidities. Second, this study highlights the advantage of LDCT by revealing incidental abnormalities in Asian populations. Previous studies have focused on Caucasian populations 10, 13. Third, we used a low threshold of positive LDCT screening because, in daily practice, the referred pulmonologists frequently treat patients with small-sized nodules. Fourth, we analyzed the role of concomitant biomarker tests, including CEA and CA 19-9 levels.

### Incidence of lung cancer

This study reports a 1.5% lung cancer detection rate at Year 1, which is similar to the 0.9% to 1.5% reported in previous reports 5-8, 10. Three studies with less than 1-year follow-up showed a 1% to 1.4% lung cancer detection rate in Taiwan 7, 8 and 1.5% in the United States 10. In the first round of screening, the lung cancer detection rates were 270/26309 (1.0%) and 56/6309 (0.9%) in the NLST and NELSON trial, respectively 5, 6.

The overall lung cancer detection rate in the BUILT study was 2.1% in the 5-year study period. In NLST, with a median follow-up duration of 6.5 years, LDCT screening detected 2.5% (649/26309) of patients with lung cancer 5. In the NELSON trial, all subjects completed a 10-year follow-up; LDCT screening detected 3.2% (203/6309) of patients with lung cancer 6. The mean length of follow-up was 0.4 (566.6/1502) years in the BLUIT study, leading to a lower rate of overall lung cancer detection.

The BUILT study period was 5 years, but the median follow-up duration was short (1 day) because of the study design. The follow-up duration in the BUILT study did not include the duration of clinical follow-up (Figure 2). For subjects who received only one LDCT and maintained clinical follow-up, the follow-up length was 1 day. Because more than 50% of the participants received only one LDCT scan during the study period, this resulted in a very short median follow-up duration (1 day). In the NLST and NELSON studies 5, 6, the follow-up length included the duration of clinical follow-up. This led to a higher cumulative incidence of lung cancer in the BUILT study (54.7 cases per 1000 person-years) than in the NLST and NELSON trial (6.45 and 5.58, respectively) 5, 6. The purely “radiological” follow-up design enhances the power of lung cancer detection in the BUILT study.

### Applicable follow-up length

According to the risk categories, the Fleischner Society recommends that the duration of follow-up should range from no follow-up to 5 years 14, while Lung CT Screening Reporting & Data System (Lung-RADS) 15, suggests that it could be up to 3 months to every 12 months. The optimal follow-up duration for lung nodules remains controversial.

In the BULIT study, all lung cancers were detected within 4 years. In Japan, Sawada et al. reported that among the 39 lung cancers (all < 3 cm) with tumor growth detected, 38 (97%) were detected within 4 years, while 1 was detected by the 9th year 16. During the 6.5-year follow-up of the NLST and 10-year follow-up of the NELSON trial, there was a steady increase in the number of lung cancers detected by LDCT 5, 6. Thus, a minimum follow-up period of 4 years is appropriate for suspicious lung nodules.

### Risk factors of lung cancer

The BUILT study showed that a family history of lung cancer in the first-degree relatives is a risk factor for lung cancer. Family history of lung cancer is a well-established risk factor for lung cancer 8, 17-19. In the United States, Cannon-Albright et al. demonstrated that a family history of lung cancer in the first-, second-, or third-degree relatives was a risk factor for lung cancer 18. In China, individuals with a family history of lung cancer in the first-degree relatives, especially the maternal side, are associated with an increased risk of developing lung cancer 17. Another study in Taiwan (Wu et al., 2016) reported that a family history of lung cancer in the first-degree or second-degree relatives was a risk factor for lung cancer 8. In addition, a family history of extrapulmonary cancers was also a risk factor for lung cancer in the Chinese population 8, 17. A similar trend was observed in the BUILT study demonstrating that a family history of extrapulmonary cancers increased the risk of lung cancer. A large-scale study is warranted to confirm the role of family history of extrapulmonary malignancy in lung cancer.

Few studies have evaluated the role of common biomarkers, such as CEA and CA 19-9, in LDCT-based lung cancer screening. In the BUILT study, an elevated CEA level was a significant risk factor for lung cancer in a population of 91% never-smokers. To our knowledge, this is the first LDCT-based lung cancer screening study to demonstrate CEA as a potential risk factor for lung cancer in “presumed low-risk” populations. A previous prospective study in Thailand enrolled purely heavy smokers and indicated that CEA in combination with LDCT could significantly enhance diagnostic performance compared to that with LDCT alone 20. In Asian populations, routine CEA testing can be considered into LDCT-based lung cancer screening programs. More large-scale prospective studies are needed to elucidate the application of serum CEA testing in lung cancer screening.

### Incidental LDCT findings

The BUILT study reported incidental abnormalities with clinical relevance, including coronary artery calcification, emphysema, and renal cell carcinoma (0.1%). In NLST, LDCT detected clinically significant extrapulmonary findings in 20% of the participants 13. In Lung Cancer Screening Demonstration Project (LCSDP), 40% of the participants had incidental findings that required further management; emphysema and coronary artery calcification were the most common findings 10. Coronary artery calcification is an established predictor of ischemic heart disease 21. Prior studies revealed that the leading cause of death was cardiovascular disease rather than lung cancer in LDCT group (NLST: 26% vs. 23%; NELSON trial: 22% vs. 18%) 5, 6. Lung nodules detected in the background of an emphysematous lung may have a higher chance of malignancy 19. Incidental findings, including coronary artery calcification and emphysema, could provide valuable information for clinicians.

LDCT has the potential to detect malignancy from the lower neck to the upper abdomen. In NLST, LDCT detected extrapulmonary malignancies in 0.4% of the participants; malignancies in the order of incidence were kidney, thyroid, and liver cancers 13. Because LDCT scans involve radiation exposure and delicate health care facilities, routine incorporation of extrapulmonary abnormalities may improve the cost-effectiveness of lung cancer screening. For extrapulmonary abnormalities, a standardized guideline for systemic reporting and management recommendations is needed in future research.

### Guidelines for lung cancer screening

There are well-established guidelines for lung cancer screening. These standards include the International Early Lung Cancer Action Program (I-ELCAP) 22, National Comprehensive Cancer Network (NCCN) guidelines for lung cancer screening 23, American College of Chest Physicians (ACCP) guidelines 24, Lung CT Screening Reporting & Data System (Lung-RADS) 15, and European guidelines 25. These guidelines provide elaborate and meticulous protocols. However, implementation of these guidelines is challenging even among resource-rich health care systems. In non-research settings, the adherence to screening protocol was 55% 26. However, following a validated guideline can help reduce unnecessary invasive procedures and follow-up examinations. Uniform and implementation-friendly lung cancer screening guidelines are strongly needed for future research. To date, shared decision-making 27 plays an important role in the trade-offs between benefits and adverse effects in the real-world setting.

The BULIT study does not provide structured protocols. The multidisciplinary Lung Cancer Committee of Taipei Tzu Chi Hospital, including pulmonologists, thoracic surgeons, thoracic radiologists, and pathologists, provides recommendations for the management of indeterminate nodules. The final decision relies on shared decision-making 27 with patients. If lung nodules that are suspected to be malignant are detected, Taiwan's National Health Insurance provides overall coverage for the subsequent medical expense, including follow-up CT, diagnostic procedures, surgical procedures, and associated hospitalization. Under relatively low financial burden, patients tend to feel comfortable to seek medical treatment.

## Limitations

Our study has some limitations. First, the sample size was relatively small, and the study design was retrospective. Second, we lacked a uniform guideline to follow up the participants, and some participants were lost to follow-up on their own will. Thus, the actual number of patients with lung cancer was underestimated. Third, this study did not provide data on outpatient follow-up. For group C, we defined the end of follow-up as the last chest CT scan before August 31, 2020. The follow-up duration depended on “radiological follow-up,” and not “clinical follow-up.” However, some individuals underwent only the baseline LDCT and were followed up at outpatient clinics for lung nodules. Fourth, we did not grade the severity of incidental findings. Any visually detected abnormalities were regarded as positive. Currently, standardized reporting and management recommendation guidelines for incidental LDCT findings are still lacking.

## Conclusions

In the 5-year BUILT study, LDCT detected lung cancer in 2.1% of the participants. A minimum of 4-year follow-up is required to detect all patients with lung cancer. We found that a family history of lung cancer and abnormal serum CEA levels were risk factors for lung cancer. Purely radiological follow-up by LDCT enhances lung cancer detection compared to that with clinical follow-up. Further studies are required to elucidate the optimal length of follow-up and role of biomarkers in the setting of LDCT-based lung cancer screening.

## Figures and Tables

**Table 1:** Baseline characteristics of all subjects and univariate logistic regression for risk factors of lung cancer

Characteristics	Lung cancer confirmed = Group A, n = 31 (2.1%)	Lung cancer not-confirmed = Groups B + C, n = 1471 (97.9%)	*P* value	Total, n = 1502 (100%)	Odds ratio (95% CI)	*P* value
**Sex, n (%)**						
Female	21 (67.7%)	822 (55.9%)	0.188	843 (56.1%)	1.658 (0.775-3.545)	0.192
Male	10 (32.3%)	649 (44.1%)		659 (43.9%)		
**Age (years), median (IQR)**	60 (52.5-65)	57 (51-64)	0.1402	57 (51-64)	1.031 (0.994-1.070)	0.104
**Subjects aged < 40 years, n (%)**	1 (3.2%)	77 (5.2%)	1.000	78 (5.2%)	0.603 (0.081-4.484)	0.622
**Subjects aged < 50 years, n (%)**	2 (6.5%)	296 (20.1%)	0.067	298 (19.8%)	0.274 (0.065-1.154)	0.078
**Ever smokers, n (%)**	4 (12.9%)	128 (8.7%)	0.343	132 (8.8%)	1.554 (0.536-4.512)	0.417
Current smokers, n (%)	1 (3.2%)	41 (2.8%)	0.673	42 (2.8%)	not applicable	
Ex-smokers, n (%)	3 (9.7%)	87 (5.9%)		90 (6.0%)		
Never smokers, n (%)	27 (87.1%)	1343 (91.3%)		1370 (91.2%)		
**Family history of lung cancer, n (%)**	5 (16.1%)	29 (2.0%)	<0.001*	34 (2.3%)	9.562 (3.430-26.66)	< 0.001*
**Family history of other cancer, n (%)**	3 (9.7%)	79 (5.4%)	0.2374	82 (5.5%)	1.888 (0.562-6.344)	0.304
**Number of Nodules, median (IQR)**	2 (1-2)	0 (0-0)	<0.001*	0 (0-0)	1.678 (1.410-1.997)	< 0.001*
**A positive result in the baseline LDCT**	31 (100%)	339 (23.0%)	<0.001*	370 (24.6%)	Cannot be estimated	0.987
**A positive result in the baseline CXR**	7 (22.6%)	18 (1.2%)	<0.001*	25 (1.7%)	23.54 (8.999-61.60)	< 0.001*
**Number of CT scans until diagnosis, median (IQR)**	2 (1-2)	1 (1-1)	<0.001*	1 (1-1)	1.924 (1.497-2.474)	< 0.001*
**Time to diagnosis (days), median (IQR)**	113 (21-628)	1 (1-1)	<0.001*	1 (1-1)	1.001 (1.000-1.002)	0.016*
**Baseline LDCT incidental findings, n (%)**						
Any incidental findings	7 (22.6%)	264 (17.9%)	0.507	271 (18.0%)	1.333 (0.569-3.127)	0.508
Bronchiectasis	1 (3.2%)	51 (3.5%)	1.000	52 (3.5%)	0.928 (0.124-6.940)	0.942
Emphysema	4 (12.9%)	83 (5.6%)	1.000	87 (5.8%)	2.477 (0.847-7.246)	0.098
Fibrocalcified lesions compatible with old pulmonary tuberculosis	0 (0%)	42 (2.9%)	1.000	42 (2.8%)	Cannot be estimated	0.998
Coronary artery calcification	3 (9.7%)	129 (8.8%)	0.749	132 (8.8%)	1.115 (0.334-3.717)	0.860
Extrapulmonary Malignancy	0 (0%)	1 (0.1%)	1.000	1 (0.1%)	Cannot be estimated	0.999
**Serum CEA ≥ 5 ng/mL, n (%)**	3 (9.7%)	23 (1.6%)	0.015*	26 (1.7%)	6.745 (1.914-23.78)	0.003*
**Serum CA199 ≥ 37 U/mL, n (%)**	1 (3.2%)	44 (3.0%)	0.614	45 (3.0%)	1.081 (0.144-8.107)	0.940
**Comorbidities**						
History of extrapulmonary malignancy	1 (3.2%)	31 (2.1%)	0.491	32 (2.1%)	1.548 (0.205 - 11.72)	0.672
Arrhythmia	0 (0%)	42 (2.9%)	1.000	42 (2.8%)	Cannot be estimated	0.998
Heart failure	0 (0%)	17 (1.2%)	1.000	17 (1.1%)	Cannot be estimated	0.999
Ischemic heart disease	0 (0%)	37 (2.5%)	1.000	37 (2.5%)	Cannot be estimated	0.998
Old stroke	0 (0%)	9 (0.6%)	1.000	9 (0.6%)	Cannot be estimated	0.999
Parkinsonism	0 (0%)	3 (0.2%)	1.000	3 (0.2%)	Cannot be estimated	0.999
Hypothyroidism	0 (0%)	10 (0.7%)	1.000	10 (0.7%)	Cannot be estimated	0.999
Hyperthyroidism	0 (0%)	12 (0.8%)	1.000	12 (0.8%)	Cannot be estimated	0.999
Autoimmune disease	0 (0%)	19 (1.3%)	1.000	19 (1.3%)	Cannot be estimated	0.998
Liver cirrhosis	0 (0%)	4 (0.3%)	1.000	4 (0.3 %)	Cannot be estimated	0.999
Chronic hepatitis	1 (3.2%)	104 (7.1%)	0.720	105 (7.0%)	0.438 (0.059-3.245)	0.419
Peptic ulcer	2 (6.5%)	145 (9.8%)	0.762	147 (9.8%)	0.631 (0.149-2.670)	0.531
End stage renal disease	0 (0%)	3 (0.2%)	1.000	3 (0.2%)	Cannot be estimated	0.999
Asthma	1 (3.2%)	24 (1.6%)	0.409	25 (1.7%)	2.010 (0.263-15.35)	0.501
Chronic obstructive pulmonary disease	2 (6.5%)	35 (2.4%)	0.176	37 (2.5%)	2.830 (0.650-12.33)	0.166
Diabetes mellitus	4 (12.9%)	131 (8.9%)	0.354	135 (9.0%)	1.515 (0.522-4.397)	0.444
Hypertension	3 (9.7%)	227 (15.4%)	0.612	230 (15.3%)	0.587 (0.177-1.948)	0.384

Categorical variables are expressed as numbers (percentages). Continuous variables with non-Gaussian distribution are expressed as medians (1^st^ quartile - 3^rd^ quartile). Cannot be estimated: the odds ratio is omitted because sparse data bias leads to inflation or shrinkage. *Denotes P<0.05. Abbreviations: IQR, interquartile range; CA 19-9, carbohydrate antigen 19-9; CEA, carcinoembryonic antigen.

**Table 2:** Characteristics, treatments, and 3-year survival rate of patients with detected lung cancer

Characteristics	n = 31
**Pathology, n (%)**	
Invasive adenocarcinoma	25 (80.6%)
Minimal invasive adenocarcinoma	2 (6.5%)
Adenocarcinoma *in situ*	2 (6.5%)
Small cell carcinoma	1 (3.2%)
Pulmonary MALT^+^ lymphoma	1 (3.2%)
**Time to diagnosis (days), median (IQR)**	113 (21-628)
**8^th^ edition of AJCC lung cancer staging, (lymphoma excluded), n (%)**	30 (100%)
0	2 (6.7%)
I	24 (80%)
II	0 (0%)
III	2 (6.7%)
IV	2 (6.7%)
Multiple primary lung cancer	2 (6.7%)
**3-year progression-free survival rate, n (%)**	28 (90.3%)
**3-year overall survival rate, n (%)**	29 (93.5%)
**EGFR mutation of adenocarcinoma, n (%)**	29 (100%)
L858R	12 (41.4%)
Exon 19 deletion	3 (10.3%)
Wild type	8 (27.6%)
Unknown	6 (20.7%)
**CT attenuation, n (%)**	
GGO	12 (38.7%)
Part-solid	8 (25.8%)
Solid	11 (35.5%)
**Size (mm), median (IQR)**	20 (10 - 25)
**First-line treatments, n (%)**	
Lobectomy	16 (51.6%)
Segmentectomy	9 (29.0%)
Wedge	4 (12.9%)
Chemotherapy	2 (6.5%)
**Subsequent treatments, n (%)**	
Observation only	26 (83.9%)
Chemotherapy	3 (9.7%)
Sequential chemo-radiotherapy	1 (3.2%)
Target therapy (afatinib)	1 (3.2%)

Abbreviations: AJCC, American Joint Committee on Cancer; EGFR, epidermal growth factor receptor; GGO, ground glass opacity; IQR, Interquartile range; MALT, Mucosa-associated lymphoid tissue.

**Table 3:** Diagnostic performance

Diagnostic performance	LDCT	CXR	CEA	CA 19-9
Sensitivity	100%	22.6%	9.7%	3.2%
Specificity	77.0%	98.8%	98.4%	97.0%
Positive predictive value	8.4%	28.0%	11.5%	2.2%
Negative predictive value	100%	98.4%	98.1%	97.9%
Area under the ROC curve	0.885	0.607	0.541	0.501

Abbreviations: LDCT, low-dose computed tomography; CXR, chest X-ray, CEA, carcinoembryonic antigen; CA 19-9, carbohydrate antigen 19-9; ROC, receiver operating characteristic.
